# Perceptions of organizational culture among non-patient-facing health system employees

**DOI:** 10.1108/JHOM-05-2024-0197

**Published:** 2025-01-31

**Authors:** Teray Johnson, Mark Newman, Sameh Shamroukh

**Affiliations:** Harrisburg University of Science and Technology, Harrisburg, Pennsylvania, USA; University of North Texas, Denton, Texas, USA

**Keywords:** Organizational culture, Health systems, Hospitals, Non-patient-facing employees, Clinical staff, Non-clinical staff, Mixed-methods, Interviews, Surveys, United States

## Abstract

**Purpose:**

The significance of organizational culture (OC) pervades all workplaces, extending even to health systems. While numerous studies have examined the perceptions of OC among nurses and physicians, there is a notable gap in understanding the perspectives of non-patient-facing health system employees. This study aims to fill this void by investigating the perceptions and drivers of OC among non-patient-facing personnel within health systems.

**Design/methodology/approach:**

This study employed a mixed-methods approach, starting with a 31-question survey disseminated to health system employees through trade organizations to capture diverse perspectives on OC. Subsequently, employees were invited to participate in semi-structured interviews. A total of 23 interviews were conducted to explore the underlying factors shaping employees’ perceptions of OC.

**Findings:**

A total of 67 surveys were completed, with 61 used in the analysis. The results revealed a predominantly positive outlook, highlighting the significance of supportive leadership and involvement in decision-making processes. The qualitative analysis identified four key themes: effective communication and transparency, coordinated teamwork, supportive leadership and the impact of external factors like the coronavirus disease 2019 (COVID-19) pandemic.

**Practical implications:**

Effective leadership should prioritize open communication, employee autonomy and involvement in decision-making. These strategies foster a culture of trust, accountability and engagement, enhancing employee morale and job satisfaction while promoting a collaborative and innovative work environment conducive to long-term success and growth.

**Originality/value:**

This research examines the often-overlooked perspectives of non-patient-facing health system staff, providing valuable insights and strategies for leaders to improve OC and create a more positive, inclusive and supportive work environment.

## Introduction

Organizational culture (OC) is a part of every workplace. Schein (2010) defines OC using three levels. The first level consists of tangible artifacts, which are visible and observable structures, processes and behaviors (Schein, 2010). The second level comprises the organization’s values and beliefs, which includes shared goals, aspirations and ideologies. The third level is composed of implicit assumptions that an organization’s employees unconsciously hold. Their assumptions influence their behavior. For this study, Schein’s definition of OC is used due to its basis in multiple research studies. In addition, Schein critiques the perception of OC as only shared values and beliefs, which results in a conception of OC as a fixed and set of values that may be problematically operationalized in management tools to change the culture. Due to the influence of OC on employee behavior and performance, attempting to create a strong OC is imperative for organizations’ leaders. Leadership has been defined as influencing others, setting examples and a vision, collaborating, and supporting and caring for others (Eddy *et al.*, 2023; Lee and Ding, 2024). Moreover, OC may have been influenced by the coronavirus disease 2019 (COVID-19) pandemic, which occurred from March 2020 through May 2023 and resulted in most businesses, commerce, schools and other activities of daily living temporarily closing (United Nations, 2023). However, hospitals remained open for care; personnel, including non-patient-facing employees, did not work remotely as a result, unlike employees in other industries. OC may have been significantly influenced by the pandemic. For example, the pandemic may have changed OC in hospitals to be more agile; decisions that may have taken several months to be made may have been made in only weeks. Moreover, smaller teams may have been assembled for quicker decision-making instead of large committees that commonly meet monthly, which is how health systems traditionally have made decisions. The pandemic may also have transformed OC to be more collaborative, supportive and prioritizing mental health (Carlasare *et al.*, 2024; Maple *et al.*, 2024). However, the pandemic may have had negative effects on OC, such as creating a more stressful, high-paced and hectic work environment (Zink *et al.*, 2024). However, the previous studies focus on physicians and nurses instead of non-patient-facing employees. It is unknown how OC has affected non-patient-facing health system employees.

OC has been studied in several industries, including healthcare (Deal and Kennedy, 1982; Braithwaite *et al.*, 2017; Prodromou and Papageorgiou, 2022; Al-Nammari *et al.*, 2023; Sen *et al*., 2023). However, little research has been conducted about perceptions of OC among non-patient-facing health system employees. Health systems are defined as “collaborative healthcare organizations encompassing hospitals, physician practices, ambulatory sites, and administrative offices,” such as information technology (AHRQ, 2023; Johnson and Shamroukh, 2024). While patient-facing employees, such as nurses and physicians, comprise a substantial percentage of health system employees and can be directly tied to a hospital’s revenue (e.g. the more patients are treated, the more revenue the health system generates), non-patient-facing employees are essential to supporting patient-facing employees. For example, data analysts are necessary to create dashboards that show which units have the most patients at certain times of the day so that nurse managers can staff those units with an adequate number of nurses at the right time. Doing so ensures that patients are cared for in a timely manner without sacrificing care quality and protects against nursing burnout resulting from staffing shortages. Additionally, environmental service employees thoroughly clean the hospitals to protect them from the spread of infections, which result in patient complications and longer lengths-of-stay. Longer lengths-of-stay prevent other patients who need care from being admitted to the hospital. If patients cannot receive necessary care in a timely manner, then they may return to the hospital sicker than they were previously. More complex patients require more resources (e.g. physicians and lab tests) would need to be used.

If non-patient-facing employees have a negative view of their health system OC, then they may be more likely to stop working for that health system (Du *et al.*, 2024; Li *et al.*, 2024). Without non-patient-facing employees supporting the patient-facing roles, nurses, physicians and other patient-facing employees will have to use their time completing these supporting tasks, such as cleaning and transporting patients to different units instead of caring for patients. Time used on tasks other than patient care has been shown to dissatisfy patient-facing employees and contribute to burnout (Gidwani *et al.*, 2017; McCormick *et al.*, 2018; Abda *et al.*, 2023). Therefore, this study examines perceptions of OC among mainly health system non-patient-facing employees, who are defined as employees who work in non-clinical areas, such as in the information systems department. They do not medically treat patients as part of their daily work – that is, they are non-patient-facing. Examples of non-patient-facing employees include non-patient-facing assistants, financial analysts and directors of marketing. Moreover, they include employees in leadership roles, such as managers and directors, and individual contributors, such as marketing associates and data analysts. Non-patient-facing employees comprise nearly half of the health system workforce; their work significantly affects hospital performance (Hospitals - May 2022 OEWS Industry-Specific Occupational Employment and Wage Estimates, 2023). This research is novel because it is one of the first to study perceptions of OC among non-patient-facing employees in health systems using a survey and semi-structured interviews to determine drivers and the context of OC for non-patient-facing health system employees.

Executive leaders have been shown to be key influencers of OC (Montminy, 2022; Mrayyan *et al.*, 2023). For example, managers promoting patient safety through their expectations and actions was shown to highly impact the safety culture of a health system (Hata *et al.*, 2022). Safety culture is defined as how employees perceive “the necessity of values, attitudes, skills and behaviors focusing on an institution’s patient care processes and the related workforce” (Sendlhofer *et al*., 2015; Özer and Şantaş, 2020). Safety culture includes “the units, organizational structures and systems that produce shared values of important issues,” beliefs about how organizational problems are addressed, and behavioral norms that contribute to patient safety (Ulrich and Kear, 2014). OC is related to safety culture; the more positively employees perceive their OC, then the more likely that a safety culture will be prominent (Juanda *et al*., 2024). An OC of empowerment has also been shown to improve patient safety culture (Rusdi *et al*., 2024). Additionally, managerial support for certain policies, such as health equity policies, can create an OC in which health equity is highly valued, which is another way in which leaders impact OC (Erasmus *et al.*, 2017). However, inadequate leadership can create an OC of a lack of “owning” problems, territorialism, cynicism and dysfunction (Dalmas and Azzopardi, 2019).

Hata *et al*. found that teamwork affects perceptions of safety culture (Hata *et al.*, 2022). Due to a lack of teamwork, there was a lack of a patient safety culture; employees did not refer patients to different departments, even when doing so was best for the patient. Therefore, the organization had a culture of not putting its patients first (Agom *et al.*, 2020). Role competition, complexity and ambiguity may reduce teamwork.

Hata *et al*. (2022) also concluded that the number of hours worked per week influences perceptions of OC, which aligns with Tlili *et al*.’s findings that a higher workload results in more negative perceptions of OC (Tlili *et al.*, 2020). Furthermore, high workloads can hinder a culture of innovation and change; employees with high workloads do not believe that change can positively affect everyone (Dalmas and Azzopardi, 2019). Their studies show several of the drivers of perceptions of OC, such as workload, leadership and teamwork. Thus, one of the objectives of this study is to assess whether these same drivers influence OC among non-patient-facing employees.

Several studies measure health system employees’ perceptions of OC. Chesley (2020) used the Competing Values Framework (CVF) to quantitatively measure and categorize different OCs in their study about OC among merged health systems in the United States. Chesley found that frontline staff, middle management and executive leadership had different perceptions of the CVF’s different categories of OC, such as team culture and hierarchal culture. However, the authors did not review in-depth the specific scores and differences between each category. Therefore, to address the complexities of OC, a quantitative and qualitative instrument to measure different aspects of OC, which includes teamwork and employee involvement, could be used.

Similar to Chesley, Mesfin *et al*. (2020) used the CVF to measure physicians’, nurses’, technical employees’ and support employees’ perceptions of OC in four Ethiopian hospitals (Mesfin *et al.*, 2020). Most perceived their hospitals to have a hierarchal culture, defined as a clear organizational structure and rules, procedures and clearly defined responsibilities; and a market culture, in which the hospital was externally oriented instead of internally oriented. However, most of their sample was comprised of patient-facing employees instead of non-patient-facing staff. The measuring of perceptions of OC among multiple job categories in a hospital demonstrates the importance of measuring patient-facing and non-patient-facing employees’ perceptions of OC among health systems, not only one hospital. Additionally, their study demonstrates that the theories of OC, such as hierarchical and market theories, apply to different job categories. This study assesses these dynamics of OC among non-patient-facing employees to continue the studies that Chesley and others began.

In their analysis of OC, particularly regarding patient safety, in Japanese health systems, Hata *et al*. (2022) found that physicians and nurses believed that the OC of their hospitals were positive, such as having a high degree of teamwork in units and an organization oriented toward continuous improvement, while unit secretaries found the OC as non-positive. In a United States’ Veterans’ Association hospital’s mental health department, perceptions of OC differed among different professions. For example, rehabilitation counselors perceived OC more positively than social workers (Sieracki *et al.*, 2024). Part of the discrepancy was regarding a lack of recognition toward clinical and mental health staff, such as social workers and physicians. Moreover, various ways of communication with different professions also may contribute to perceptions of whether OC is transparent. For example, a daily huddle in which the same information is provided to different professions simultaneously, such as safety and staffing needs, increases whether employees perceive their OC as psychologically safe to share concerns, may increase perceptions of transparency and communication, and contribute to a culture of feedback in which leaders consider employees’ insights. Their work suggests that different categories of employees perceive culture differently. However, these studies do not address perceptions of OC among different non-patient-facing roles; moreover, few studies focus on different perceptions of OC among different roles. While several studies assess perceptions of OC among different professions, the researchers aggregate the data and do not indicate the differences between different professions’ perceptions of OC, nor are they thoroughly discussed (Jafari *et al.*, 2020; Dziurka *et al.*, 2022). Thus, this study seeks to measure how employees with different roles perceive OC.

A paucity of studies exist in which non-patient-facing health system employees are interviewed and surveyed about their perceptions of OC. Most of the recent literature in healthcare focuses on perceptions of OC among patient-facing employees in health systems instead of perceptions of non-patient-facing employees. Since OC can influence behavior and work performance among all employees, further research is needed about the perceptions of OC among non-patient-facing employees (Simpson *et al.*, 2014; van Rooij and Fine, 2018). The behavior and work performance of non-patient-facing employees could impact patient care. For example, a high workload contributes to a stressful OC. Therefore, non-patient-facing schedulers who schedule appointments for patients from lab orders may not do so in a timely manner due to high workloads preventing them from scheduling all needed appointments in a workday. Patients may not receive timely care, which results in worsened patient outcomes, such as complications of sicknesses. The more positively an employee views his or her OC, the better his or her work performance could be. OC affects factors beyond patient care, such as turnover. Employees who work in a hierarchical OC, characterized by a low degree of employee autonomy and managers controlling how employees work and a low amount of managerial support are more likely to resign from their workplace (Jung *et al*., 2017; D’Alessandro-Lowe *et al.*, 2024; Du *et al.*, 2024; Li *et al.*, 2024). Turnover has financial consequences for organizations; recruiting, training and hiring new employees is a substantial expense in time and labor (Lee *et al*., 2021). For example, nursing turnover costs hospitals approximately $88,000 per nurse (Brook *et al.*, 2019; Bae, 2022; Xie *et al.*, 2024).

Because OC changes depending on departments, leaders, and various other factors, qualitative and quantitative studies could be conducted to address cultural dynamics (Muhr *et al*., 2022). Additionally, cultural dynamics could be accounted for by integrating the differentiation theory of OC in which the differences of subcultures within an organization are accounted for. Interviews among the same employees in the same organizations, or interviewees’ assessments of how leaders view other teams and subcultures, such as ethnic minorities, add to overall perceptions of OC.

A void in the literature exists in which non-patient-facing health system employees have not been interviewed or surveyed about their perceptions of their organization’s culture. Most studies focus on patient-facing employees or do not utilize a mixed-methods approach but are cross-sectional (Roth *et al.*, 2021; He *et al.*, 2023; Logroño *et al.*, 2023). This study fills the literature gap of perceptions of OC among non-patient-facing health system employees by interviewing and surveying them about their perceptions of their organization’s culture during the COVID-19 pandemic. Previous studies have not focused on the pandemic’s influence on non-patient-facing employees’ perceptions of OC, even though they may have been affected by resource constraints, such as a lack of staff, as patient-facing employees were. This study shows the importance of measuring and accounting for non-patient-facing employees when leaders cultivate OC through their actions and decisions.

To further explore health system non-patient-facing employees’ perceptions of OC, the following research questions will be explored:

RQ1. What are non-patient-facing health system employees’ perceptions of their health systems’ OC?

RQ2. What are the drivers of OC (e.g. strength of teamwork, leadership support and workload levels) among non-patient-facing health system employees?

### Research methodology

This research examines the perceptions of OC among mainly non-patient-facing health system employees.

### Setting, measurement and study design

This is an explanatory sequential study in which a quantitative phase was first implemented, which involved survey data collection and analysis, followed by a qualitative phase, which encompassed interviews, interview recording and transcription, and coding to develop themes.

In the quantitative phase, Kovner *et al*.’s reliable and valid 31-item survey measuring organizational aspects that led to turnover was used (Kovner *et al.*, 2007a). Table 1 shows how their survey items and subdomains relate to aspects of OC in the literature; moreover, many of the questions used in the survey were derived from scales measuring aspects of OC, such as procedural justice, as stated by Ashkanasay *et al*. (2011). Previous research in which their survey was used on non-nurses showed a reliability score of 0.97 and a validity score of 0.87 (Johnson and Newman, 2024). Their survey instrument measures factors of OC, such as the level of managerial support and teamwork in an organization via a Likert scale that measures items from 1 (poor) to 5 (excellent). The subdomains of each category are supervisor support (6 items), work-group cohesion (6 items), work attitudes (12 items) and safety (7 items). Each of these subdomains reflects aspects of OC. For example, perceptions of supervisor support, co-worker cohesion and attitudes reflect whether the OC is supportive or encourages collaboration (Ogbonnaya *et al*., 2018; Montminy, 2022; Ahmed and Bein, 2023). Employee attitudes also affect behavior, which, thus, influences OC (Schein, 2010; Montminy, 2022). The remaining six items pertained to demographics. Further information about these domains, including the items in each, is detailed elsewhere (Johnson and Shamroukh, 2024).

Kovner *et al*.’s study aimed to estimate turnover rates among newly licensed registered nurses (RNs) using a survey measuring aspects of OC, such as teamwork and perceptions of supervisor support. Questions were adapted from their survey to use in the current study and address the research question of how non-patient-facing health system employees perceive their OC. Furthermore, based on previously cited literature, factors such as teamwork and leadership support impact perceptions of OC; Kovner *et al*.’s survey questions address these influencers of OC. Moreover, Kovner *et al*.’s survey questions are derived from several instruments measuring aspects of OC (Johnson and Newman, 2024). Their survey questions also align with questions in the Organizational Culture Assessment Questionnaire (OCAQ), which is used for the general population of employees. Demographics questions were added to assess differences between locations, employees’ positions, health system sizes, employees’ departments and ownership status – that is, who owns the health system, such as the United States’ federal government; the public, which pays taxes to support the health system; or a private party or private equity investor (Welch *et al.*, 2023).

The survey was hosted on Link to the website, and responses were collected and participants recruited from February 2022 to October 2022. Participants could complete the survey in any location because the survey was hosted online.

Following the quantitative phase, a qualitative phase was implemented to assess the nuances within and deepen understanding of the survey results. The qualitative phase involved similar questions as the quantitative phase but allowed the ability to ask more probing questions to understand the reasoning behind several survey responses, such as whether respondents believed that their OC was positive. An interview guide is included as a Supplementary Table.

### Data collection

In the quantitative phase, an appropriate sample size for adequate statistical power in a 95% confidence interval was determined to be 385 based on an estimated population of 2,000,000 health system workers. purposeful sampling was used in which all employees of subacute health systems were invited to participate via trade organizations (organizations that advocate and provide informational content for certain industries) and social media (e.g. LinkedIn). The survey was shared via people of influence (e.g. manager-level and above) in the first author’s (TJ) social network, who included managers of health systems, who then distributed the Link to the website survey link to their contacts to minimize bias. All responses were confidential and anonymous. Participants provided informed consent at the beginning of the survey. The survey was sent to over 100 trade organizations and health systems; however, only 67 responses were received due to several trade organizations and health systems either failing to respond to the first author or declining participation for diverse reasons (Johnson and Shamroukh, 2024). In the solicitation message, a definition of non-patient-facing employees was included. Responses involving patient-facing employees, such as bedside nurses, were excluded from the sample to focus exclusively on non-patient-facing roles. However, non-patient-facing clinical staff, including nurse and physician managers, were retained in the study to ensure their perspectives were represented in the analysis. In the qualitative phase, a convenient, purposeful sample of potential interviewees was contacted via e-mail or social media, such as LinkedIn messages and Reddit posts on communities targeting non-patient-facing health system employees. Additionally, the author received interviewee recommendations from colleagues within the American College of Healthcare Executives (ACHE), which is a trade organization for healthcare professionals. At the end of the survey, participants were asked to optionally provide their e-mail address if they wanted to be contacted for an interview. To be included in the study, participants must be employees of a hospital or health system (defined as having at least one hospital instead of only another type of healthcare facility, such as primary care offices) and non-patient-facing in their daily tasks (e.g. business intelligence developers or nurses who worked as managers and did not clinically treat patients as part of their daily work). Interested interviewees were invited to participate in a virtual interview that occurred over Microsoft Teams or phone. The interviews were recorded and transcribed for accuracy. Transcriptions and recordings were entered into ATLAS.ti 22 for analysis.

In the qualitative phase, the first author invited employees who worked in health systems located in the Mid-Atlantic United States to participate in a one-on-one interview about their perceptions of OC via LinkedIn and e-mail. The first author conducted semi-structured interviews until data saturation was reached, for a total of 23 interviews from 23 individuals. Data saturation was determined to have been reached when no new information or themes were obtained (Guest *et al*., 2020). Triangulation was achieved via using a mixed-methods approach; the interview responses deepened understanding of the survey results. Additionally, the interviews were conducted among different non-patient-facing employees in different health systems; for example, business intelligence developers and non-patient-facing assistants were interviewed. Their responses were similar, which corroborated the results. Therefore, the results are more likely to be generalizable. Moreover, fidelity was ensured by recording and transcribing the interviews word-by-word. The interviewer also asked participants if her interpretation of their responses was correct, which is known as member-checking.

The interviews were conducted from October 2022 to January 2023 via Microsoft Teams and phone. Verbal and written consent was obtained prior to the interviews. The interviews were recorded and transcribed using Microsoft Teams. The interviewer used an interview guide from the questions and dimensions of the OCAQ to ask participants open-ended questions (Sashkin and Rosenbach, 1996). Each interview lasted approximately 30- to 45-min. If further clarification was required, then probing questions were asked. The interviewer uploaded the recordings and transcriptions into ATLAS.ti 22 (a qualitative data analysis software package) for emergent coding.

This research has been approved by Harrisburg University of Science and Technology’s Institutional Review Board (IRB# 20221026).

### Analysis

The quantitative survey results were analyzed using R. Because all survey questions were mandatory to answer, there were no missing data to address. The means of each question response were found, and correlation analysis of statistically significant correlations was conducted to assess trends and relationships between scores. Moreover, each question addresses a driver of OC, such as leadership support and teamwork.

**Figure 1:**
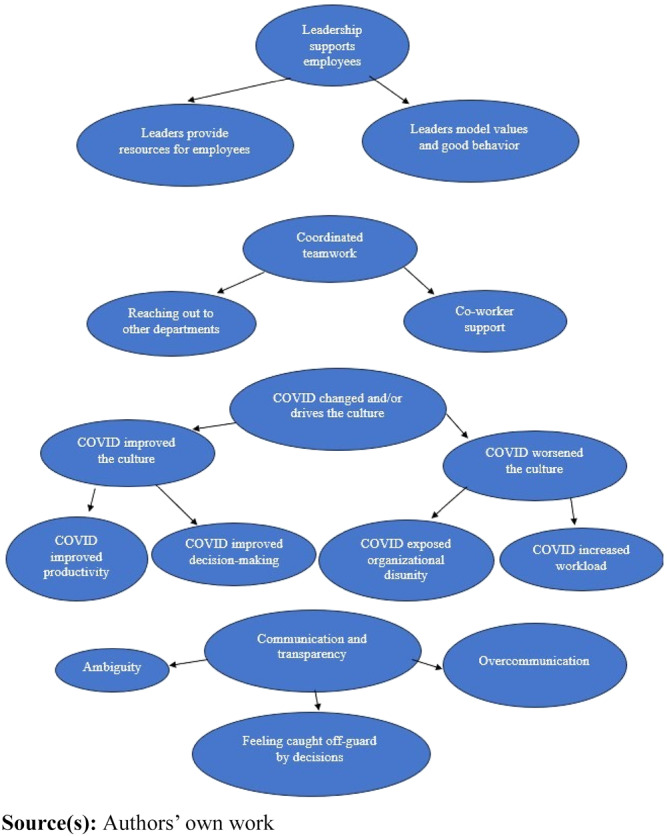
Coding tree

The interview transcriptions were analyzed using ATLAS.ti 22. The analytical categories, or codes, were derived emergently (inductively) in which the transcriptions were first read and then codes were created from the transcriptions line-by-line. The codes were identified as recurring, similar responses to questions and were based on the values coding method, in which codes were created that reflected participants’ worldviews and placed in a codebook. The codes were refined iteratively based on the themes discovered in the interviews. The codes were arranged into categories; the process continued until all codes were sorted into categories. Themes were identified from the categories by assessing the most common responses to questions, such as the COVID-19 pandemic influencing the organization’s culture. The themes and codes were repeatedly checked against the transcriptions to ensure an accurate representation of interviewees’ responses. For example, if several interviewees repeatedly mentioned “lack of leadership support” in their responses, then “lack of leadership support” was created as a code and then categorized into the theme, “Interviewees perceived a lack of leadership support.” To validate the interviews, the interviewer conducted member-checking in which she asked clarifying questions to verify that her interpretation of each interviewee’s response was correct. Additionally, a pilot interview was conducted to validate the questions. Confirmability was achieved through peer debriefing in which the interview questions and interview settings were assessed by co-authors and four members of the research team prior to interviewing the participants (McMahon and Winch, 2018). The co-authors and research team thoroughly reviewed the transcripts to verify that the assigned categories accurately reflected the quotes identified by the first author during the coding and interpretation process. Following the interviews, the team recorded memos and documented their reflections to capture insights and contextual understanding. Reliability was ensured by reviewing and correcting the transcriptions, defining the codes, continually comparing data with the codes, and writing memos about the codes and their definitions. The coding tree is shown in Figure 1.

## Findings

### Quantitative phase

During the quantitative phase of our study, a robust response was garnered, with a total of 67 healthcare personnel actively participating in the survey. This ensured a comprehensive dataset characterized by completeness, as each question necessitated a response, thereby eliminating any instances of missing values from the results.

To ensure the integrity and relevance of the data, responses from individuals not affiliated with non-patient-facing health systems were meticulously filtered out. This exclusion encompassed various roles, including consultants, non-managerial registered nurses employed in clinical departments and board members, thus refining the dataset to focus solely on pertinent participants.

The demographic makeup of the respondents, as delineated in Tables 2 and 3, offers valuable insights into the composition of our sample. Notably, the predominant occupational category among participants was administration, with a substantial count of 20 individuals. Within this administrative cohort, the roles of administrators and assistant administrators were prevalent, comprising both middle- and senior-level management personnel, with a combined count of 18 individuals.

**Table 2:** Survey respondents’ departments

Department	Number of respondents
Administration	20
Board of directors	4
Community health	3
Diagnostic imaging/radiology	1
ED	4
Inpatient psychiatry	1
Inpatient surgery (management)	1
Lab Services	1
Med/surg (management)	2
Pharmacy	1
Quality and patient safety	4
Analytics	4
Case management/social work	1
Finance	1
Graduate medical education	1
Information technology	2
Marketing/business development	2
Operations	1
Strategy	1
Patient experience	1
Radiation oncology	2
Refused to answer	3

**Note(s):** For clinical departments (e.g. inpatient psychiatry, med/surg), interviewees were manager-level or above and did not perform patient care as part of their daily role

**Source(s):** Authors’ own work

**Table 3:** Survey respondents’ positions

Position title	Number of respondents
Administrator/assistant administrator/supervisor	18
Strategy consultants	3
Case manager	1
Clerical	1
IT support	2
Medical assistant	12
Medical doctor (MD)	10
Patient coordinator/access representative	3
Chaplain	1
Physician chief/chair	1
Project manager	1
Nursing administrator	4
Residency coordinator	1
Other	2

**Source(s):** Authors’ own work

An examination of Tables 4–7 sheds light on additional demographic characteristics of the participants. It emerges that a considerable proportion of respondents were affiliated with non-profit health systems boasting substantial capacities, specifically those with 500 beds or more. Additionally, the majority of participants were situated in urban locales, underscoring the urban-centric nature of our sample.

**Table 4:** Survey respondents’ health systems’ total hospital size

Bed size	Number of respondents
6–24 beds	4
25–49 beds	0
50–99 beds	3
100–199 beds	7
200–299 beds	3
300–399 beds	5
400–499 beds	6
500 beds or more	33

**Source(s):** Authors’ own work

**Table 5:** Survey respondents’ health systems’ geographic location

Location	Number of respondents
Rural	22
Urban	38
Suburban	1

**Source(s):** Authors’ own work

**Table 6:** Survey respondents’ health system ownership status

Type	Number of respondents
Federal	2
For-profit	19
Non-profit	40

**Source(s):** Authors’ own work

**Table 7:** Interviewees’ departments

Department	Number of respondents
Administration	4
Ambulatory care	3
Community health	3
Radiation oncology	1
Maternal/women’s and child health	2
Philanthropy	1
Physical therapy	1
Analytics	2
Marketing	1
Operations	1
Human resources	1
Radiation oncology	1
Quality and patient safety/performance improvement	2

**Source(s):** Authors’ own work

This meticulous curation and analysis of participant demographics provide a nuanced understanding of the sample composition, enriching the subsequent interpretation and generalizability of our study findings. The mean scores of the survey responses that assess the drivers of OC are shown in Table 8. Most respondents believed that their supervisors strongly supported staff. For example, respondents reported that their supervisors listened to job-related difficulties (mean = 4.07). Additionally, respondents reported that their supervisors inspire them to perform their best (mean = 3.69). A correlation analysis revealed that supervisors’ inspiring respondents to perform their best, providing honest feedback to their employees and listening to job-related difficulties were positively associated with respondents believing that the overall culture of their organization was positive (*r* = 0.57, *r* = 0.59, and *r* = 0.42 respectively) and respondents stating that employees were involved in decisions that affected their work (*r* = 0.59, *r* = 0.32, and *r* = 0.52 respectively).

**Table 8:** Means and standard deviations

Survey item	Mean	SD
I show concern for my co-workers' well-being	4.67	0.51
My co-workers show concern for my well-being	4.16	1.04
I am satisfied with my job	3.92	1.11
My direct supervisor pays attention to what I’m saying	4.02	1.23
My supervisor listens to job-related difficulties	4.07	1.21
My supervisor encourages those he/she supervises to express their opinions	4.02	1.20
I have a good chance to be promoted	3.36	1.20
I have opportunities to do a number of different things	4.12	1.05
Members of my work unit/department are involved in decisions that directly affect their work	3.75	1.14
Decisions are made based on research, data and technical criteria, as opposed to political concerns	3.59	1.17
I think that my present employer is a great organization to work for	3.98	1.23
My supervisor and other leaders inspire me to perform my best	3.69	1.15
I am friendly toward my co-workers	4.51	0.62
My co-workers are friendly toward me	4.34	0.81
I show my co-workers how to work successfully	4.18	0.85
My co-workers show me how to work successfully	4.31	0.90
I give honest feedback to my co-workers	4.12	0.81
My co-workers and supervisors give me honest feedback	4.12	0.88
Patient and employee safety are never sacrificed to get more work done	3.74	1.24
I have the level of technology needed to perform my job effectively and safely	4.07	1.11
I have the supplies and equipment needed to perform my job effectively and safely	4.23	1.04
As an employee, I feel comfortable reporting potential or actual patient and employee safety problems	4.25	1.01
Our procedures and systems are good at preventing errors from happening	3.85	1.01
I participate in quality improvement processes, such as root cause analyses	4.00	1.20
A patient is likely to receive high-quality care on my unit (or hospital in general if not patient-facing)	4.13	1.04
My unit/department often works as a team to improve processes or systems of care as a result of errors that were reported in my unit (or hospital in general if not patient-facing)	4.18	0.96
I effectively carry out my roles and responsibilities	4.54	0.72
My co-workers use appropriate language with patients, family and visitors	4.48	0.70
I use appropriate language with patients, family and visitors	4.74	0.63
Overall, the culture of the hospital is positive	3.92	1.10
Overall, the culture of the organization helps to make sure that patients are given high-quality care	4.12	1.05

**Source(s):** Authors’ own work

Many respondents also believed that their cultures showed strong patient orientation, in alignment with the customer orientation dimension of the OCAQ, and as demonstrated by the mean score of respondents believing that their OC is conducive to providing high-quality patient care is 4.12. Furthermore, most respondents self-reported that they and their co-workers use appropriate language with patients, family and visitors (mean = 4.48 for co-workers using appropriate language and 4.74 for self-reports of using appropriate language with visitors). Respondents believed that patients were likely to receive high-quality care in their health system (mean = 4.13). The correlation analysis revealed that having all the supplies and equipment needed to perform one’s job was positively associated with respondents’ believing that patients received high-quality care in their health system (*r* = 0.60).

Most respondents believed that they and other employees were somewhat involved in decisions that directly affected their work (mean = 3.75). The correlation analysis revealed that the aforementioned score was positively associated with respondents believing that their employer is great to work for, the overall culture of the organization is positive, and that employees work as a team to improve processes or systems (*r* = 0.37, *r* = 0.35 and *r* = 0.48 respectively).

Standard deviations for each response ranged about 1 point for each response, as shown in Table 8. The significant spread between responses could be due to the variety of respondents from different health systems and positions.

### Interviews

A total of 23 non-patient-facing health system employees participated in the interviews that were conducted to further assess the drivers of OC. Table 9 provides an overview of the interviewees’ job positions. Based on the responses, four themes were found.

**Table 9:** Interviewees’ positions

Position	Number of respondents
Associate director	1
COO	2
Divisional director	1
Quality coordinator	2
Vice president	1
Non-patient-facing assistant	2
Assistant manager	1
Administrator	1
Director of quality	1
Director of ambulatory services	1
Senior director of marketing	1
Business intelligence developer	1
Manager of data analytics	1
Vice president of human resources	1
Manager/director of community health	2
Director of women’s and children’s health	1
Director of nursing	1
Lean specialist	1
Director of operations	1

**Source(s):** Authors’ own work

### Theme 1: communication and transparency

One theme was communication, which was necessary to inform employees, especially during the COVID-19 pandemic and other times of change. One human resources vice president stated:

We do a really decent job at communicating to folks, especially as it relates to change. If I use COVID as an example, there was a heck of a lot of communication. I think the unfortunate piece with COVID is that it wasn't static and so you could have put something out on Monday that said, Hey, look, you're good. No mask is required. And then as things evolved it was like, holy cow, not only do you need a mask, but it needs to be an N-95, and it needs to be worn all the time. And so being able to communicate and pivot quickly was really, really important.

During the pandemic, conflicting information came from different sources, such as the news and the internet. Interviewees who were leaders stated that they communicated frequently and, perhaps, overcommunicated to provide clarity to both employees and patients. A senior director of marketing stated that the health system CEO created several weekly videos that were published on social media platforms to inform patients and address their anxieties and concerns. The CEO also communicated frequently to employees.

However, non-executive (i.e. director-level and below) interviewees believed that communication could be lacking or ambiguous, causing perceptions of a lack of transparency and honesty. Because of the lack of communication, several interviewees stated that they felt caught off-guard by decisions from executive leadership. For example, one director of women’s and children’s services felt caught off-guard when she was informed of a hiring freeze one day before it was implemented. A data analytics manager shared the following when direct reports could be laid off due to the pandemic’s economic effects:

I saw that [economic recovery plan] meeting on my calendar […] 5:00 o'clock on Thursday and [my direct reports] were laid off on a Friday. […] The day before, I got a meeting on my calendar for the next day. […] It was 6:30 at night. And I was talking to [my supervisors] [and] the vice-president, and asking them what this meeting was all about the next day and why I didn't know more detail and arguing with the fact that one of my people was gonna be laid off and they were not telling me who it was gonna be. […] And [the vice-president] still didn't want to tell me even until Friday. And I was like, are you kidding me? I can't believe you're not gonna tell me. Like, who? This affects my life, my everyday work. And you're not gonna tell me who and what the reasoning is, and what all happened behind this decision or whatever. […] I felt like I was definitely blindsided by that.

Both the manager and other employees conveyed concerns regarding insufficient communication and transparency from executive leadership. Despite executive leaders’ perception of effective communication and a transparent culture within the organization, non-executive staff held differing views. They felt that while they understood the need for some information to be withheld, they desired greater transparency from their executive leaders. Non-executive employees perceived that executive leaders tended to conceal matters within their health systems, particularly within the OC.

### Theme 2: coordinated teamwork

A second theme was coordinated teamwork among departments to adapt to changes, alleviate workloads and accomplish organizational goals. Several interviewees stated that they felt comfortable asking other departments for help and that employees in other departments often asked them for help. Additionally, some health systems used their employees creatively during staffing crises. For example, when a hospital in one health system lacked pharmacists, they asked pharmacists from other hospitals to assist. In contrast, one director of the women’s and children’s service line stated that, when there was a lack of staffing in the health system practices, the practices did not receive assistance from other departments. The lack of staff throughout that health system caused a lack of willingness to work as a team across departments, while teamwork within the department remained strong.

According to various interviewees, opinions diverged on the extent of frontline and non-managerial staff involvement in decision-making processes. While some interviewees acknowledged existing involvement, others perceived potential for increased engagement to foster a culture of teamwork. A director of operations emphasized the benefits of employee involvement in decision-making, citing improved employee support for challenging organizational changes. Interviewees highlighted the importance of feeling valued through participation in decisions, with the establishment of committees serving as a mechanism for soliciting employee input. Moreover, several non-managerial interviewees expressed a keen interest in actively participating in decision-making processes.

### Theme 3: leadership supports employees

A third theme was leadership support. Interviewees described their leadership supporting employees in several ways, such as by providing childcare during the COVID-19 pandemic and encouraging staff to use their vacation time. Other interviewees stated that leaders advocate for their employees. A non-patient-facing assistant witnessed a situation in which an employee on medical leave was going to be discharged from the organization. The chief nursing officer persuaded other executive leaders to not discharge the employee. Advocacy for employees was reiterated by a quality coordinator in another health system:

I have spoken to her about just how I feel with things culturally … And one thing that I do notice, I feel she has really been trying to, you know, at least get my name out there and […] give me my credit where credit is due, which I can appreciate.

However, the same interviewee expressed the lack of support from other levels of leadership:

It just can't be one person that [advocates for employees]. It’s not just me … There are so many other people within the organization that are like me that are not getting credit for the work that they're doing, that aren't being recognized, that deserve to be directors and executive directors and board members that are not because they're being kept in a box.

Some interviewees expressed feelings of being overlooked in terms of their work contributions and opportunities for advancement. While several health systems boasted formal recognition programs, certain interviewees felt their efforts were not duly acknowledged by leadership. For instance, a director within the women’s and children’s service line found themselves burdened with additional responsibilities without receiving a formal promotion or salary increase, prompting them to explore alternative job prospects.

Nevertheless, the prevailing sentiment among both managerial and non-managerial interviewees was one of perceived support from their leaders. Many leaders were observed to prioritize work-life balance and actively address employees' anxieties and apprehensions. For instance, a vice president overseeing ancillary services noticed that employees were accumulating unused vacation hours and encouraged them to take time off, emphasizing the importance of self-care. Furthermore, leaders initiated formal programs aimed at supporting employee well-being, such as providing lunch, designated relaxation areas and implementing shortened workweeks. Additionally, several interviewees noted the accessibility of their leaders, highlighting their readiness to provide assistance whenever needed.

### Theme 4: the COVID-19 pandemic changed and/or drives the culture

In some instances, the COVID-19 pandemic changed the culture. Several interviewees stated that the pandemic changed how their health systems function. In one health system, the pandemic created a culture of isolation and a lack of teamwork for several units, such as the intensive care unit (ICU). Interviewees noticed that employees who worked in the ICU felt isolated from other departments; other employees did not visit that unit due to fear of COVID-19 exposure.

In some health systems, the pandemic improved support and teamwork. A director of operations stated that, at the beginning of the pandemic, employees “rallied” to support patient-facing clinicians. Moreover, when one employee was infected with COVID-19, another employee used vacation time to care for the sick employee’s children. When directors were infected, executives would lead those directors’ departments until the directors returned.

While the pandemic improved organizational strengths, it also exposed negative OCs. A director of ambulatory care expressed that the pandemic showed how the health system’s inflexibility would no longer suffice in everyday operations. Furthermore, the pandemic exacerbated staffing and workload issues at several health systems. One data analytics manager stated that in departments that were understaffed prior to the pandemic, including the manager’s own department, the manager and other employees became more overwhelmed as their workloads increased. Fewer people did the same jobs or had tasks added without increased compensation or promotions. Two other employees at the same health system expressed similar sentiments; workload increased due to resignations and workforce reductions. A non-patient-facing assistant at another health system stated that the pandemic exacerbated the existing toxic culture, such as leadership not following through on their commitments or listening to their staff.

In some cases, the pandemic improved the culture. A non-patient-facing assistant stated that, when employees began to work remotely, the environment lost its “anxiety-building, oppressive, rigorous” energy. The office became quiet and peaceful. Furthermore, a director of nursing stated that when pandemic-related issues prevented nurses from providing patient care, leaders addressed those issues immediately due to focus on the pandemic. In that aspect, the pandemic increased leadership support. Several interviewees stated that leaders made decisions more quickly because information regarding the COVID-19 pandemic changed quickly, whereas prior to the pandemic, the same decisions would have taken months to make and implement. Interviewees also expressed that meeting virtually instead of in person increased organizations’ agility, especially regarding decision-making.

### Integrations between survey and interview responses

The survey results and interview responses are integrated in several ways. For example, most survey respondents believed their OC to be positive. However, several interviewees expressed dissatisfaction with their organization’s OC, such as one interviewee stating that the workplace was “toxic.” Furthermore, several interviewees expressed dissatisfaction with communication; decisions caught several by surprise, or they did not receive communication about decisions such as position eliminations.

The survey results showed that most respondents felt comfortable reporting potential safety issues. The interviews revealed a nuance in which individual contributors (i.e. non-managers) expressed distrust of leadership, which resulted in their not speaking to their organization’s leaders about non-patient- and employee-safety-related issues. For example, several expressed beliefs of co-workers and leaders harboring unconscious bias that caused a lack of promotional opportunities. In contrast to the individual contributors’ responses, most survey respondents and interviewees who were manager-level and above believed that their leaders and supervisors were supportive and listened to job-related issues. Interviewees who were individual contributors expressed that, if they did speak about job-related issues to their supervisors, their concerns were not addressed. The interviews showed that managers often felt as though they could not control the issues of which their direct reports spoke due to organizational limitations, such as budget constraints. The contrast between individual contributors’ and leaders’ responses revealed a lack of understanding of what individual contributors believed leaders could change and influence. Oftentimes, leaders wanted to address their employees’ concerns, such as one manager who attempted to secure a monetary raise and promotion for one direct report. However, organizational constraints prevented leaders from doing so. The constraints sometimes resulted in turnover, such as one leader who stated that two individual contributors resigned from a health system due to a lack of promotion because they did not have a university degree.

The survey respondents indicated that they had sufficient technology, supplies and equipment to perform their jobs. The interviews revealed that employees believed that they were understaffed due to a high workload or position eliminations resulting in additional tasks being given. While the interviewees believed that they had adequate technology, equipment and supplies, they did not believe that they had a sufficient amount of staff.

## Discussion

The qualitative findings elucidate the pivotal role of communication and transparency in shaping employees' perceptions of OC within health systems. Virtually all interviewees underscored the paramount importance of effective communication and transparency, highlighting their multifaceted benefits in fostering a positive work environment. Notably, frequent communication channels were instrumental in assuaging employee anxiety, mitigating feelings of uncertainty and curbing the proliferation of rumors within the workplace. While the survey did not directly inquire about communication practices, the recurring emphasis on communication in interviews, coupled with insights from the OCAQ, suggests a significant association between communication effectiveness and OC.

Previous research has delineated contrasting OCs characterized by communication patterns, ranging from cultures of silence to cultures of voice (Bogosian, 2018; Sashkin and Rosenbach, 1996; Hower *et al.*, 2020). The absence of clear communication channels, particularly during periods of financial instability or organizational crises, engendered feelings of apprehension and uncertainty among interviewees. Instances, where strategic plans were withheld or inadequately communicated, exacerbated concerns among employees, especially amidst workforce reductions or furloughs during the pandemic. Such lapses in communication precipitated a toxic work environment typified by fear and anxiety, underscoring the deleterious impact of inadequate communication practices on OC (Wakefield *et al.*, 2016; Machen *et al.*, 2019; Kreitzer *et al*., 2020). A culture of transparency reduces anxiety and, therefore, promotes a more peaceful work environment (Sun *et al.*, 2023).

Conversely, the qualitative analysis unveiled the pivotal role of teamwork in shaping positive perceptions of OC among employees. Survey results corroborated this, demonstrating a statistically significant positive correlation between teamwork and perceptions of a positive OC across departments. Many interviewees attested to the emphasis placed on teamwork within their health systems, with interdepartmental collaboration being a prevalent practice. Such collaborative endeavors fostered a culture conducive to feedback and safety, wherein every employee’s contribution was valued (Ferguson *et al.*, 2022). However, staffing constraints posed significant challenges to teamwork, with understaffed departments struggling to extend support to one another amidst heightened workloads. This underscores the intricate interplay between staffing levels and teamwork dynamics within healthcare organizations, necessitating further exploration in future research endeavors. The American Association of Critical-Care Nurses found that a healthy OC includes adequate staffing and teamwork among nurses (Pun *et al.*, 2022). Pun *et al*.’s study also found that teamwork positively influences OC among nurses. The results of this study show that the same results are true for non-patient-facing employees.

Being understaffed may have contributed to negative perceptions of OC among interviewees. A non-patient-facing assistant expressed that the health system was chronically understaffed; the lack of staff resulted in low employee morale. A stressful OC was cultivated in which there was a continual sense of urgency. One study found that understaffing contributed to a negative safety culture among nurses but did not study the effects of understaffing among non-patient-facing personnel (Imes *et al.*, 2023). This study shows that understaffing affects non-patient-facing personnel. Therefore, leaders should be attentive to resolving staffing issues among non-patient-facing and patient-facing personnel. However, the non-patient-facing assistant stated that employees tried to help one another, despite the lack of staff, showing a high degree of teamwork. Further research is needed to determine the relationship between staffing and teamwork.

Moreover, the survey findings elucidated the salience of supervisor support in shaping employees’ perceptions of OC. Positive correlations were observed between perceptions of a positive OC and various dimensions of supervisor support, echoing findings from prior studies highlighting the pivotal role of organizational support in fostering a psychologically safe work environment (Iqbal *et al.*, 2021; Sjöblom *et al*., 2022). The qualitative insights underscored the significance of supervisor advocacy in cultivating a culture of recognition and value among employees. However, instances where advocacy failed to translate into tangible outcomes, such as promotions or recognition, underscored the potential pitfalls of unmet expectations and their implications for employee turnover (Lee *et al.*, 2018; Choudhuri, 2022).

Furthermore, the qualitative analysis shed light on the impact of the COVID-19 pandemic on OC within health systems. External forces, such as the pandemic, exerted profound influences on OC, with interviewees attesting to both positive and negative ramifications. While the pandemic laid bare existing issues, such as overwork and communication deficits, it also catalyzed positive cultural shifts, such as enhanced collaboration, organizational support and empathy among employees (Al-Shamali *et al.*, 2022; Shamsudin and Velmurugan, 2022). Leaders’ proactive measures, including improved communication strategies, remote work allowances and initiatives to alleviate employee burdens, emerged as critical factors in mitigating adverse effects and fostering a resilient OC amidst crisis situations (Zhang *et al.*, 2022; Zhou and Zhang, 2022).

Survey results reflected employee attitudes that affect OC, such as whether employees showed concern for co-workers’ well-being and friendliness toward peers. Most respondents believed that their co-workers showed concern. Further aspects of perceived organizational support, such as supervisors listening to their employees, were reflected in high survey scores in the support-related questions. The literature also shows that supervisors listening to employees and showing concern for employees’ well-being shape OC (Buick *et al.*, 2024; Wang *et al.*, 2024). However, little literature studies how co-workers’ attitudes toward peers shape and reflect OC. The survey results contrast to the literature in studying how co-worker attitudes toward one another shape OC.

In addition, the survey results reflect moral behavior and OC; for example, most respondents believed that they and their co-workers speak appropriately with visitors and patients, which is moral. Moreover, most respondents believed that safety was never sacrificed to complete more work. Caring for the client (i.e. patients and visitors) demonstrates an organization’s morality (Nijsmans, 1991). Furthermore, having an ethical climate in which employee and patient safety are not sacrificed for performance and achieving goals contributes to perceptions of OC (Thomas *et al.*, 2024). Data-driven decisions instead of decisions driven by politics also demonstrate morality and organizational justice in which organizations do what is best for employees and patients instead of doing what is best to achieve only their goals (Lotfi-Bejestani *et al.*, 2023). The literature shows that perceptions of organizational fairness affect whether employees view their organization’s culture as just (Lotfi-Bejestani *et al.*, 2023). Studies also find that supervisors’ acting justly affects employees’ perceptions of OC; while the survey results and interviews show that data-driven decision-making and treating patients and visitors appropriately are two aspects of justice. One study demonstrates how supervisors’ behavior affects perceptions of OC (Skarlicki *et al.*, 2016). Supervisors have substantial influence over OC, which then affects employee behavior. For example, supervisors including employees in decision-making and allowing employees to express their opinions contribute to an inclusive OC (Ashikali, 2023). The survey results reflect that most employees believed that their supervisors did both. In contrast to this study’s results, Ashikali (2023) finds that most employees do not believe that they are included in decision-making. The interviewees similarly stated that they were often not included in decision-making.

In summary, the qualitative findings underscore the multifaceted nature of OC within health systems, highlighting the critical role of communication, teamwork, supervisor support and external influences in shaping employees' perceptions and experiences within the workplace. These insights provide valuable implications for organizational leaders seeking to cultivate a positive and resilient OC amidst dynamic and challenging environments.

## Conclusion

The findings from both the survey outcomes and interview insights underscore the critical importance of fostering a congenial, inclusive and supportive OC within health systems, extending beyond the realms of nursing and medical personnel to encompass non-patient-facing staff. It is evident that the prevailing OC significantly influences the job satisfaction and retention rates of non-patient-facing employees. Consequently, the cultivation of a robust OC emerges as a pivotal strategy for health systems to mitigate turnover rates and the associated costs and time investments incurred in replacing employees.

Moreover, this study sheds light on the convergence of perceptions regarding OC among different echelons within health system hierarchies. Both frontline contributors and managerial personnel alike grapple with the challenges posed by prevailing cultural dynamics, such as pervasive overwork, particularly exacerbated amidst the exigencies of the pandemic. Conversely, executive-level stakeholders tend to espouse more sanguine views regarding communication efficacy, whereas non-executive employees express reservations about the clarity and transparency of communication channels within their respective health systems.

Efforts aimed at enhancing OC must prioritize expeditious and transparent communication practices, ensuring that changes, objectives, and strategies are conveyed promptly and comprehensively to all employees. Addressing prevailing concerns regarding OC, including communication gaps and staffing inadequacies, is paramount to fostering a culture of trust, collaboration and mutual support within health systems. Such interventions not only bolster organizational resilience but also contribute to bolstering employee morale and organizational cohesion.

### Research limitations

Despite its valuable contributions to both literature and the healthcare sector, this study faces several notable limitations that warrant acknowledgment and consideration. Firstly, the lack of generalizability of qualitative findings arises from the restriction of interviews with employees within health systems located specifically in the Mid-Atlantic region of the United States. To enhance the breadth and applicability of future research, it is advisable to conduct interviews with employees from a more diverse array of health systems across the entire nation.

Secondly, the absence of certain demographic information, including details regarding race/ethnicity and age, was deliberate to encourage survey participation and safeguard respondent anonymity. However, this limitation impedes a comprehensive understanding of how perceptions of OC may vary among individuals of different racial/ethnic backgrounds, age groups and other demographic categories. Hence, forthcoming studies should incorporate additional demographic questions to facilitate a more nuanced analysis of OC perceptions.

Furthermore, the quantitative phase of the study is constrained by a relatively small sample size, comprising 61 participants. Despite efforts to engage over 100 influential individuals, such as health system CEOs, COOs and board members, alongside various trade associations, a significant proportion did not respond to multiple solicitations. While this limitation was partially mitigated by conducting 23 supplementary interviews to glean insights into OC drivers and influencers, there remains an opportunity for future studies to bolster sample size through incentivization strategies, such as offering participants the chance to win a gift card upon survey completion.

While this study represents a valuable step toward understanding OC within healthcare settings, its limitations underscore the need for ongoing research endeavors aimed at broadening the scope, inclusivity and robustness of findings. By addressing these limitations, future studies can make more substantial contributions to the scholarly discourse and practical applications within the healthcare industry. Moreover, despite the attempt to minimize bias, interviewees were contacted via the first authors’ connections. Therefore, there may have been bias in the selection of interviewees.

### Research implications

This research contributes to the literature by examining the perceptions of OC among mainly non-patient-facing health systems employees. Prior research has focused on mainly nurses and physicians. However, this research shows the importance and influence of OC on non-patient-facing employees’ work performance, turnover rates and behavior.

Additionally, this research shows the impact of the COVID-19 pandemic on perceptions of OC among non-patient-facing health system employees. Prior research has focused on the impact of the pandemic on patient-facing employees. This study shows that the pandemic changed the OC or exposed issues of the OC in health systems that non-patient-facing employees noticed, such as an increased workload and lack of work/life balance. The results show that the pandemic did not affect only patient-facing employees of health systems but also non-patient-facing employees.

The survey results validate the use of the OCAQ tool for interviewing employees about OC. The interview responses enrich the literature and research about OC regarding non-patient-facing health system employees by providing the reasoning behind survey responses. Prior research has either surveyed health system employees or interviewed the employees but has not conducted both phases by contacting the same group of employees who were contacted to distribute the survey to their networks. The multifaceted implementation of this research is new among non-patient-facing health system employees. Future studies could use a similar technique for researching perceptions of non-patient-facing health system employees regarding other subjects.

### Practical implications

This research is intended for health systems managers and executives to pinpoint opportunities for improvement that may not be highlighted in existing employee engagement surveys. Additionally, this research highlights the drivers of OC. This research has several implications for practice, such as demonstrating that transparent and open communication can improve employees’ perceptions of OC. Therefore, leaders should focus on improving communication within their health systems. Additionally, employee autonomy and including employees in decision-making cultivates an OC of teamwork. Leaders should ensure that employees are autonomous by allowing employees to complete projects independently and include them in decision-making so that they feel as though they are a part of the team. Further research should be conducted to assess whether addressing drivers of culture improves non-patient-facing employees’ perceptions of culture through a survey or follow-up interviews.

Due to the phased implementation of the study, the reasoning behind the survey responses was gathered. Leaders can use the depth of the interview responses and the interviewees’ practical advice to improve OC in their organizations. Moreover, leaders can use this phased approach when conducting yearly employee evaluation surveys; most surveys involve only a quantitative survey. However, by implementing an interview phase in which a focus group of employees is interviewed, they can gather insights beyond the survey results and, thus, return from the survey with practical ways to improve OC.

### Final remarks

OC is primarily shaped by the actions and attitudes of organizational leaders. As such, it is imperative for leaders to prioritize the development of a supportive and inclusive OC that fosters a sense of purpose among all employees. This concerted effort not only enhances employee morale but also elevates the quality of their work performance.

A positively perceived OC yields numerous benefits for healthcare organizations. By nurturing a culture where employees feel valued and included, turnover rates can be reduced, as individuals are more likely to remain committed to an organization where they feel appreciated and supported. Furthermore, a conducive OC contributes to heightened job satisfaction among employees, resulting in increased motivation and engagement levels.

Importantly, the ripple effects of a positive OC extend beyond the organization’s internal dynamics to impact patient care outcomes. When employees feel fulfilled and supported in their roles, they are more inclined to deliver high-quality care and service to patients. Consequently, enhanced patient satisfaction levels are often observed in healthcare settings characterized by a nurturing and supportive OC.

## Supplementary Table

Table A1

**Table 1:** Survey questions, factors, and sources

Survey item	Scale	Latent factor/Subscale
C1: I show concern for my co-workers' well-being	Organizational Citizenship Behavior	Interpersonal Helping
C2: My co-workers show concern for my well-being	Organizational Citizenship Behavior	Interpersonal Helping
C3: I am satisfied with my job	Michigan Organizational Assessment Questionnaire	Overall Job Satisfaction
C4: My direct supervisor pays attention to what I’m saying	Newly Licensed Registered Nurse (NLRN) Survey	Supervisory Support
C5: My supervisor listens to job-related difficulties	Organizational Citizenship Behavior/NLRN Survey a	Loyal Boosterism/Supervisory Support
C6: My supervisor encourages those he/she supervises to express their opinions	Organizational Citizenship Behavior/NLRN Survey	Loyal Boosterism/Supervisory Support
C7: I have a good chance to be promoted	Job Descriptive Index/NLRN Survey	Satisfaction with Promotion
C8: I have opportunities to do a number of different things	Job Characteristics Inventory/NLRN Survey	Variety
C9: Members of my work unit/department are involved in decisions that directly affect their work	Distributive and Procedural Justice/NLRN Survey	Procedural Justice
C10: Decisions are made based on research, data, and technical criteria, as opposed to political concerns	Distributive and Procedural Justice/NLRN Survey	Procedural Justice
C11: I think that my present employer is a great organization to work for	Psychological Attachment Instrument/NLRN Survey	Compliance/Organizational Commitment
C12: My supervisor and other leaders inspire me to perform my best	Organizational Commitment Questionnaire/NLRN Survey	Organizational Feedback/Supervisory Support
C13: I am friendly toward my co-workers	NLRN Survey	Work-group Cohesion
C14: My co-workers are friendly toward me	NLRN Survey	Work-group Cohesion
C15: I show my co-workers how to work successfully	NLRN Survey	Mentor Support
C16: My co-workers show me how to work successfully	NLRN Survey	Mentor Support
C17: I give honest feedback to my co-workers	Job Characteristics Inventory/NLRN Survey	Feedback/Mentor Support
C18: My co-workers and supervisor give me honest feedback	Job Characteristics Inventory/NLRN Survey	Feedback/Mentor Support
C19: Patient and employee safety are never sacrificed to get more work done	Hospital Survey on Patient Safety Culture (HSOPSC)/NLRN Survey	Staffing and Work Pace/Quantitative Workload
C20: I have the level of technology needed to perform my job effectively and safely	NLRN Survey	Organizational Constraints
C21: I have the supplies and equipment needed to perform my job effectively and safely	NLRN Survey	Organizational Constraints
C22: As an employee, I feel comfortable reporting potential or actual patient and employee safety problems	Hospital Survey on Patient Safety Culture (HSOPSC)	Communication Openness
C23: Our procedures and systems are good at preventing errors from happening	Hospital Survey on Patient Safety Culture (HSOPSC)/Procedural Justice	Overall Perceptions of Patient Safety/Procedural Justice
C24: I participate in quality improvement processes, such as root cause analyses	Distributive and Procedural Justice/NLRN Survey	Procedural Justice
C25: A patient is likely to receive high-quality care on my unit (or hospital in general if not patient-facing)	Distributive and Procedural Justice/NLRN Survey	Procedural Justice
C26: My unit/department often works as a team to improve processes or systems of care as a result of errors that were reported in my unit (or hospital in general if not patient-facing)	NLRN Survey	Work-group Cohesion
C27: I effectively carry out my roles and responsibilities	Nursing Culture Assessment Tool (NCAT)/NLRN Survey	Behaviors/Autonomy
C28: My co-workers use appropriate language with patients, family and visitors	NCAT/NLRN Survey	Communication/Organizational Constraints
C29 - I use appropriate language with patients, family and visitors	NCAT/NLRN Survey	Communication/Organizational Constraints
C30: Overall, the culture of the hospital is positive	NCAT/NLRN Survey	Satisfaction/Organizational Constraints
C31: Overall, the culture of the organization helps to make sure that patients are given high-quality care	NCAT/NLRN Survey	Satisfaction/Organizational Constraints

**Source(s):** Ashkanasy *et al*. (2011), Kovner *et al.* (2007b), Kennerly *et al*. (2012)

**Table A1:** Interview guide

Topics/Dimensions	Questions
Demographics/Background	Tell me about yourself What department do you work in? How long have you been working in healthcare? How long have you been working in your current health system? What motivated you to start working in healthcare?
Perceptions of organizational culture	How would you describe your health system’s culture? What do you love about working here?
Ability to manage change	How do your health system’s leaders, from executives to your direct supervisor, address unexpected change? How did your health system’s leaders address the COVID-19 pandemic? Are new ideas encouraged and implemented?
Coordinated teamwork	In general, do people work as an effective team inside and outside your department? Do people regularly reach out to teammates outside their department for help?
Patient orientation	Are staff encouraged to quickly and excellently respond to patients’ needs? If so, do leaders set the example – how? In general, do both clinical/patient-facing and non-clinical/non-patient-facing staff place a high priority on meeting patients’ needs?
Recognition	Are employees regularly recognized and thanked for their work efforts, new ideas or accomplishing departmental or individual goals/milestones (e.g. decreasing hospital-acquired infections and obtaining a degree)? Are efforts and accomplishments informally recognized?
Cultural strength	Do you generally feel like your anxieties and concerns are heard and considered by leadership and taken into consideration? Are the objectives and priorities of the organization regularly communicated and well understood? Is information about the organization communicated in a timely manner from leaders? Have you ever felt in the dark or caught off-guard by a company decision that suddenly came and impacted your work?
Improving organizational culture	How could your health system’s leaders improve the culture here?
End of interview	Is there anything you would like to add?

**Source(s):** Authors’ own work
